# Naxitamab Activity in Neuroblastoma Cells Is Enhanced by Nanofenretinide and Nanospermidine

**DOI:** 10.3390/pharmaceutics15020648

**Published:** 2023-02-15

**Authors:** Lucrezia Galassi, Martina Rossi, Pietro Lodeserto, Monia Lenzi, Francesca Borsetti, Manuela Voltattorni, Giovanna Farruggia, Paolo Blasi, Isabella Orienti

**Affiliations:** 1 Department of Pharmacy and Biotechnology, University of Bologna, Via San Donato 19/2, 40127 Bologna, Italy; 2 Center for Applied Biomedical Research (CRBA), University of Bologna, 40126 Bologna, Italy; 3 National Institute of Biostructures and Biosystems, Via delle Medaglie d’Oro 305, 00136 Rome, Italy

**Keywords:** anti-GD2 monoclonal antibodies, CHP-134 cells, SH-SY5Y cells, fenretinide, spermidine, neuroblastoma, antitumor activity, cell motility, quantitative phase imaging, flow cytometry

## Abstract

Neuroblastoma cells highly express the disialoganglioside GD2, a tumor-associated carbohydrate antigen, which is also expressed in neurons, skin melanocytes, and peripheral nerve fibers. Immunotherapy with monoclonal anti-GD2 antibodies has a proven efficacy in clinical trials and is included in the standard treatment for children with high-risk neuroblastoma. However, the strong neuro-toxicity associated with anti-GD2 antibodies administration has hindered, until now, the possibility for dose-escalation and protracted use, thus restraining their therapeutic potential. Strategies to increase the efficacy of anti-GD2 antibodies are actively sought, with the aim to enable chronic treatments that could eradicate minimal residual disease and subsequent relapses, often occurring after treatment. Here, we report that Nanofenretinide and Nanospermidine improved the expression of GD2 in neuroblastoma cells (CHP-134) and provided different effects in combination with the anti-GD2 antibody naxitamab. In particular, Nanofenretinide significantly increased the cytotoxic effect of naxitamab while Nanospermidine inhibited cell motility at extents proportional to naxitamab concentration. In neuroblastoma cells characterized by a low and heterogeneous basal expression of GD2, such as SH-SY5Y, which may represent the cell heterogeneity in tumors after chemotherapy, both Nanofenretinide and Nanospermidine increased GD2 expression in approximately 50% of cells, thus shifting the tumor population towards improved sensitivity to anti-GD2 antibodies.

## 1. Introduction

Gangliosides are glycosphingolipids that are overexpressed on cancer cells. Most gangliosides cannot be targeted for cancer therapy because they are extensively found in healthy tissues. The ganglioside GD2, on the contrary, is strongly expressed in neuroblastoma (NB) cells, while in normal tissues its expression is limited to neurons, skin melanocytes, and peripheral nerve fibers [1]. Furthermore, GD2 is extensively expressed in all NBs of any grade and staging, making this ganglioside a valuable target for therapy by monoclonal antibodies (mAbs), CAR T Cells, or NK cells [2].

**Figure 1:**
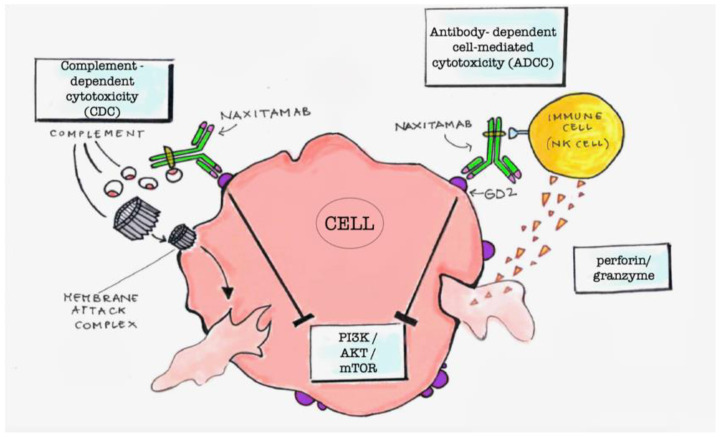
Schematic representation of cytotoxicity induced by anti-GD2 antibodies. Complement-dependent cytotoxicity (CDC) is due to the formation of the membrane attack complex following the antibody linkage with GD2 that triggers activation of the complement proteins. The membrane attack complex disrupts the cell membrane causing cell death. Antibody-dependent cell-mediated cytotoxicity (ADCC) is due to activation of immune cells such as macrophages, NK cells, or granulocytes by linkage of the Fc region of the anti-GD2 antibody with the Fcγ receptors of these cells. The Fc-Fcγ linkage triggers phagocytosis by macrophages or destruction of the tumor cells by the perforins and granzymes secreted by NK cells and granulocytes. A further mechanism is the downregulation of PI3K/Akt/mTOR signaling network triggered by the Fc- Fcγ linkage.

The linkage of anti-GD2 mAbs with the GD2 molecules exposed on the cell surface can induce cell cytotoxicity by different mechanisms [3]. The main mechanisms are the antigen-dependent cell cytotoxicity (ADCC) and the complement-dependent cytotoxicity (CDC) (Figure 1). ADCC is determined by the presence of immune cells in the tumor environment. It consists on the linkage of the Fc residues of the mAbs, linked to the GD2 molecules, with the Fcγ receptors of macrophages, NK cells, or granulocytes. This triggers phagocytosis by macrophages or destruction of the tumor cells by the perforins and granzymes that are secreted by NK cells and granulocytes [4,5]. CDC is induced through the binding of the serine protease complex C1 to the Fc domains of the mAbs linked to the GD2 molecules expressed on tumor cells. This complement pathway results in the activation of a cascade signaling, which causes the formation of the membrane attack complex with a disruption of the target cells [6]. Another mechanism is the downregulation of the PI3K/Akt/mTOR signaling network that has been demonstrated in NB cell lines upon mAbs linkage with GD2 (Figure 1) [7]. 

Naxitamab (NX) and dinutuximab are the two anti-GD2 mAbs that are currently used as immunotherapy agents in the treatment of NB. Naxitamab is a humanized anti-GD2 mAb and represents the latest bioengineering improvement. Treatment with naxitamab or dinutuximab has provided a survival increase in patients in remission and in patients with relapsed or refractory disease, but late relapses are frequently observed [8,9]. To improve efficacy, increased doses of anti-GD2 mAbs should be administered for prolonged periods of time. 

However, dose-escalation and protracted administrations are hampered by toxic effects such as the whole-body allodynia, a severe pain perceived in response to light touch, generated by the binding of anti-GD2 mAbs to normal peripheral sensory nerve fibers. Despite the use of opioids to control the pain, allodynia remains the major dose-limiting toxicity and, in addition, the use of opioids may interfere with the efficacy of antitumor treatments [10]. 

An improvement of GD2 expression on tumor cells could enhance anti-GD2 mAbs efficacy without a dose-escalation. Thus, strategies to increase GD2 expression on tumor cells or to enhance the sensitivity of tumor cells to the effects of anti-GD2 mAbs are actively sought to make tolerable doses of mAbs able to provide better clinical results [11,12,13].

The increased expression of GD2 has been largely demonstrated in NB cells by treatments with fenretinide and has been attributed to the activation of serine palmitoyltransferase, dihydroceramide synthase and sphingomyelinase as a part of the fenretinide mechanism in increasing dihydroceramide/ceramides ratio in tumor cells [14,15,16,17]. Fenretinide increased GD2 expression in NB cells characterized by low basal levels of GD2 expression such as SH-SY5Y [14] or high basal levels of GD2 expression such as CHLA-15, CHLA-20, CHLA-79 and CHLA-136 [17], demonstrating independence from the heterogeneity of GD2 expression on tumor cells.

So, with the aim to increase anti-GD2 mAbs activity, we evaluated Nanofenretinide (NF) in combination with the anti-GD2 antibody naxitamab in NB cell lines characterized by high basal GD2 expression, such as CHP-134, and low, heterogeneous basal GD2 expression, such as SH-SY5Y. 

Nanofenretinide is a nanoformulation based on phospholipid nanomicelles containing fenretinide, which demonstrated activity in a wide range of tumor cell lines and xenograft models [18,19,20,21,22]. The ability of Nanofenretinide to penetrate tumor cells and transport high drug amounts has been proven in NB cells that showed higher intracellular drug concentrations with respect to free fenretinide [21]. Therefore, treatment with Nanofenretinide is expected to amplify the ability of free fenretinide in increasing GD2 expression in NB cells. 

We also evaluated Nanospermidine (NS) in combination with naxitamab. Nanospermidine is a nanoformulation made of phospholipid nanomicelles containing spermidine. It had been previously evaluated for its ability to transport supraphysiological concentrations of spermidine into NB cells inducing an ROS increase [22]. The activation of stress-associated pathways, triggered by the ROS increase, is expected to improve the cell sensitivity to naxitamab. Indeed, improvements of anti-GD2 mAbs activity have been reported by combined treatments with drugs inducing cell stress [23]. In particular, the ability of anti-cancer drugs such as doxorubicin, topotecan, carboplatin, cisplatin, etoposide and paclitaxel to trigger stress-activated pathways, such as JNK/SAPK or PERK [24,25], has been correlated to the enhanced cytotoxicity of their combinations with anti-GD2 mAbs.

## 2. Materials and Methods

### 2.1. Chemicals

N-4-hydroxyphenyl-retinamide (fenretinide, 4-HPR) was purchased from Olon Spa (Milan, Italy); spermidine, soy L-α-phosphatidylcholine, glyceryl tributyrate, 2-hydroxypropyl beta cyclodextrin (Mw 1460) and KOH were purchased from Sigma-Aldrich (Milan, Italy), naxitamab was a kind gift from Y-mAbs Therapeutics (Horsholm, Denmark), and ethanol absolute anhydrous was purchased from Carlo Erba Reagents (Milan, Italy). DMEM High Glucose medium, RPMI 1640 medium, dichlorofluorescein diacetate (DCHFDA), Hoechst 33342, glutamine, trypsine/EDTA solutions, and Human Serum (HS) were purchased from Sigma-Aldrich Italia (Merk Life Science S.r.l. Milan, Italy).

### 2.2. Preparation of Spermidine Nanomicelles (NS) and Fenretinide Nanomicelles (NF)

Fenretinide nanomicelles were prepared according to a method previously reported [20,21]. Briefly, soy phosphatidylcholine (4 mmoles), glyceryl tributyrate (2 mmoles), 2-hydroxypropyl beta cyclodextrin (0.4 mmoles), and KOH 10 N (400 µL, 4 mmoles) were mixed to homogeneity to obtain a semisolid phase. A solution of fenretinide (1.2 mmoles) in ethanol (300 µL) and KOH 10 N (120 µL) was added to the semisolid phase and the resultant mixture was homogeneously dispersed in PBS pH 7.4 to 50 mg/mL. The coarse nanomicelle suspension obtained was filtered through 0.2 µm filters, dialyzed for 72 h (dialysis membrane Mw cutoff 10 KD) against PBS pH 7.4, and then, the dialyzed phase was finally lyophilized. The dry product was reconstituted with water to 50 mg/mL NF and stored at −22 °C until use. Spermidine nanomicelles were prepared as previously reported [22] by mixing soy phosphatidylcholine (4 mmoles), glyceryl tributyrate (2 mmoles), 2-hydroxypropyl beta cyclodextrin (0.4 mmoles), KOH 10 N (400 µL, 4 mmoles), and spermidine (2 mmoles) to homogeneity to obtain a semisolid phase that was dispersed in PBS pH 7.4 to 50 mg/mL. The coarse nanomicelle suspension obtained was filtered through 0.2 µm filters, dialyzed for 72 h (dialysis membrane Mw cutoff 10 KD) against PBS pH 7.4, and then, the dialyzed phase was lyophilized. The dry product was reconstituted with water to 50 mg/mL NS and stored at −22 °C until use. Empty nanomicelles (No) were prepared by the same procedure but without addition of drugs.

### 2.3. Characterization of the Nanomicelles

The loading of fenretinide and spermidine in the nanomicelles was evaluated as previously described [22]. Briefly, the reconstituted nanomicelle dispersions (50 mg/mL) were diluted (1:3, *v*/*v*) with an ethanol:water (1:1, *v*/*v*) mixture and analyzed for drug content in comparison with the empty nanomicelles. The content of fenretinide was obtained by UV spectroscopy (Shimadzu UV-1601) at 360 nm. Spermidine content was evaluated by fluorimetry after production of hydrogen peroxide from spermidine and reaction of hydrogen peroxide with a fluorometric probe (Ex/Em = 535/587 nm) generating a signal proportional to the polyamine concentration. The reactions were carried out by a polyamine assay kit (Merck Milan, Italy) according to the manufacturer’s instructions. Mean size, polydispersity index and zeta potential were measured at 37 °C on nanomicelle suspensions in PBS at 0.05 mg/mL (Malvern Nano-ZS Spectrometer, Malvern, UK). A minimum of 12 measurements were made per sample. The results were the combination of 3 runs of 10 min each for a total accumulation correlation function time of 30 min. The nanomicelle stability to drug leakage was measured by the release of spermidine and fenretinide from NS and NF, respectively, by dialysis at 37 °C, as previously described [20,21,22]. Briefly, the reconstituted nanomicelles suspensions (50 mg/mL) were diluted with PBS 7.4 containing 10% Human Serum (HS) to a final concentration of 0.05 mg/mL. A total of 1 mL of the diluted suspension was introduced into a release chamber separated by a dialysis membrane (Mw cutoff 5KD, Fisher Scientific) from a receiving compartment filled with 10 mL PBS pH 7.4 containing 10% HS. Leakage from the nanomicelles was determined by evaluating the concentrations of fenretinide or spermidine in the receiving phase at increasing time intervals. The concentration of fenretinide was evaluated spectrophotometrically by its maximum absorption wavelength (360 nm) and the concentration of spermidine was determined by the previously described polyamine assay kit. Sink conditions were maintained throughout the experiment.

### 2.4. Conjugation of Naxitamab with Fluoresceine

The conjugation of naxitamab with fluoresceine (NX-F) was performed by the FluoroTag FITC Conjugation kit (Merck Milan, Italy) according to the manufacturer’s instructions. Briefly, a naxitamab solution (5 mg/mL) in 0.1 M sodium carbonate-bicarbonate buffer pH 9.0 was mixed with a fluorescein isothiocyanate (FITC) solution in the same buffer at 20:1 FITC/Naxitamab molar ratio. After 2 h of incubation in the dark with gentle stirring, NX-F was isolated by a Sephadex G-25 M column. The fluorescein/protein molar ratio in the NX-F conjugate was 3.17, as determined by UV absorbance at 280 nm and 495 nm.

### 2.5. Cell Lines

SH-SY5Y and CHP-134 neuroblastoma cells were kindly provided by Prof. Giovanni Perini (Department of Pharmacy and Biotechnology, University of Bologna, Italy). For the present study, SH-SY5Y cells were grown in DMEM supplied with 10% HS, Penicillin (100 UI/mL), and Streptomycin (0.1 mg/mL) at 37 °C in a 5% CO_2_ humidified atmosphere. They were maintained in 25 cm^2^ culture flasks (Corning, Tewksbury, MA, USA) and harvested using 0.25% Trypsin in 0.2 g/L EDTA solution. CHP-134 cells were grown in RPMI 1640 supplied with 10% HS, Penicillin (100 UI/mL), and Streptomycin (0.1 mg/mL) at 37 °C in a 5% CO_2_ humidified atmosphere. They were maintained in 25 cm^2^ culture flasks (Corning, Tewksbury, MA, USA) and harvested by gentle agitation.

### 2.6. Evaluation of the Biological Effects of Nanofenretinide, Nanospermidine, and Naxitamab on Neuroblastoma Cells

#### 2.6.1. MTT Assay

To evaluate the effect of Nanofenretinide, Nanospermidine, and naxitamab on cell viability, the cells were treated with the single components or with Nanofenretinide or Nanospermidine in combination with naxitamab, and MTT assays were performed. Treatments were conducted at Nanofenretinide and Nanospermidine concentrations ranging from 0.025 to 0.20 mg/mL corresponding to fenretinide concentrations ranging from 5 to 40 μM or spermidine concentrations ranging 22 to 173 μM, respectively. The empty nanomicelles were also tested at the same concentrations as the loaded ones. Naxitamab was evaluated at 5, 10, and 20 μg/mL as a single component and in combination with 0.05 mg/mL Nanofenretinide or Nanospermidine. Cell proliferation and viability were detected by 3-(4,5-dimethylthiazol-2-yl)-2,5-diphenyltetrazolium bromide (MTT) tetrazolium salt assay. This assay is based on MTT reduction to the insoluble formazan salt by cellular dehydrogenase. The amount of formazan produced is indicative of the number of viable cells in the sample [26,27]. To perform the MTT assay, the cells were seeded at 10 × 10^3^ cell/cm^2^ in 96 multiwell plates, and, after 24 h, they were treated with Nanofenretinide, Nanospermidine, or the empty nanomicelles for 24 h, otherwise they were treated with naxitamab alone or in combination with Nanofenretinide or Nanospermidine for 24 or 72 h. Subsequently, 10 µL of MTT solution 5 mg/mL was added to each well to a final concentration of 0.5 mg/mL. After 4 h at 37 °C in the dark, 100 µL of sodium dodecylsulfate (SDS) 10% (*w*/*v*) in HCl 0.01 mM was added to each well to dissolve the purple formazan crystals and left overnight on a shaker. The absorbance was read for each well on a TECAN plate reader (Männedorf, Switzerland) at 570 nm. Absorbance was normalized by a second reading at 690 nM to avoid interference by the turbidity of biological samples.

#### 2.6.2. Quantitative Phase Imaging (QPI) Microscopy

QPI is a potent microscopic technique based on the assessment of the phase delay generated by light waves passing through cells. Holography or ptychography methods are used to convert the phase delay in pixel values within the generated image. Pixel intensity is correlated to the physical thickness and the refraction index of the cells that depend on biomolecule composition and organization [28,29,30]. In this study, we used a Lifecyte microscope (Phase Focus, Sheffield, UK) equipped with a 10 X 0.25 NA) objective to perform QPI based on ptychography. This microscope collects multiple diffraction patterns from spatially overlapping regions of the samples to form QPI images and estimate different parameters such as cell number, confluence, morphology, dry mass, motility, cell thickness, etc. Cells used for QPI analysis were seeded in a 96-well plate (Corning, Tewksbury, MA, USA) at 4 × 10^3^ per well and treated after 24 h at the same concentrations as for MTT assays. Images were acquired every 60 min for 3 days on cells kept at 37 °C and 5 % CO_2_. QPI data were processed using Cell Analysis Toolbox software (Phase Focus, Sheffield, UK). We evaluated cell confluence, thickness, cell displacement, and instantaneous velocity to assess cell vitality and spreading ability.

#### 2.6.3. Confocal Laser-Scanning Fluorescence Microscopy

Confocal laser-scanning microscopy (CLSM) is a valuable tool for imaging living and fixed specimens containing fluorophores. High-resolution images are generated by superposition of photons emitted from the fluorophore that reach the detector during one exposure period. To image with CLSM, the cells were seeded in 4-well chamber slides (Lab-Tek Chambered Coverglass, Thermo Fisher, New York, NY, USA) at 10 × 10^3^ cell/cm^2^. After 24 h, they were treated with Nanofenretinide or Nanospermidine, 0.05 mg/mL for 24 h. NX-F was subsequently added at 10 ug/mL and, after 1 h the medium, was removed and substituted with fresh one. The cells were subsequently incubated for 1 h with 1 μg/mL Hoechst 33342 to stain cell nuclei. Following cell exposure to Hoechst 33342, the cells were washed with PBS three times and fresh medium was added. As controls, the cells were exposed to an Anti-Mouse FITC antibody produced in goat (Sigma Aldrich, Milan Italy). The samples were analyzed by a confocal laser-scanning microscope (Nikon C1s), equipped with an oil immersion lens (Nikon PlanApo 40, 1.4-NA). Excitations at 405 nm and 488 nm were obtained by a diode laser and an argon laser respectively, and the emissions at 460 nm (blue) and 525 nm (green) were recorded. Image analysis was made by Image J Software (version 1.53a, U. S. National Institutes of Health, Bethesda, MD, USA). To evaluate cell death by treatment, the cells were seeded in Cellstar 96-well microplates (Greiner, AT) at 10 × 10^3^ cell/cm^2^ and, after 24 h, they were treated with NX, NF, and NS, and with the combinations NF plus NX and NS plus NX. As a comparison, the cells were also treated with empty nanomicelles (No) in combination with NX. After 72 h, calcein (2 nM) was added to spot the living cells and propidium iodide (PI) (7 μM) to stain dead cells. Specimens were analyzed using a Nikon C1s confocal laser-scanning microscope that was equipped with a Nikon PlanApo 10X. Excitation was performed at 488 nm and emissions at 525 nm and 605 nm (red) were recorded. Image analysis was made by Image J Software (version 1.53a, U. S. National Institutes of Health, Bethesda, MD, USA).

### 2.7. GD2 Surface Expression Evaluated by Flow Cytometry

To determine GD2 surface expression the cells were treated with NF, NS, free fenretinide, or free spermidine for 72 h, detached using trypsin (0.05%) with EDTA, washed in complete media, and pelleted by centrifugation. They were counted and 2.5 × 10^5^ cells were resuspended in 0.5 mL DMEM without serum and incubated with NX-F (20 μg/mL) or with a matched control mAb of irrelevant (non-biologic) specificity Anti-Mouse FITC antibody produced in goat (Sigma Aldrich, Milan, Italy) for 1 h at room temperature in the dark. 

Cells were then washed twice in PBS buffer and transferred to filter top tubes with addition of 7 nM PI to stain dead cells. Data were acquired using a BioRad S3 cell sorter, equipped with an Argon Ion Laser tuned at 488 nm, and with the emission settled, respectively, at 525 nm (green) and 605 (red). A total of 10,000 events were acquired from the live (PI negative) singlet gate for each cell line. Unstained cells of each sample were used as negative controls, and their fluorescence was placed in the first decade of the logarithmic intensity plot. Exported FCS files were then analyzed using FlowJo_v10 software and geometric mean fluorescence intensity was determined for every sample. Data were visualized as histograms of the fluorescence distribution with FlowJoTM (BD Biosciences, Franklin Lakes, NJ, USA). 

### 2.8. Statistical Analysis

All experiments were performed at least in triplicates on three independent samples. The data were processed using one-way analysis of variance (ANOVA) followed by Dunnett’s multiple comparison test. Group differences were considered significant when *p* < 0.05. Statistical analysis was carried out by GraphPad Prism Software (version 6.0c, GraphPad Software, San Diego, CA, USA).

## 3. Results

### 3.1. Characterization of NS and NF

**Figure 2:**
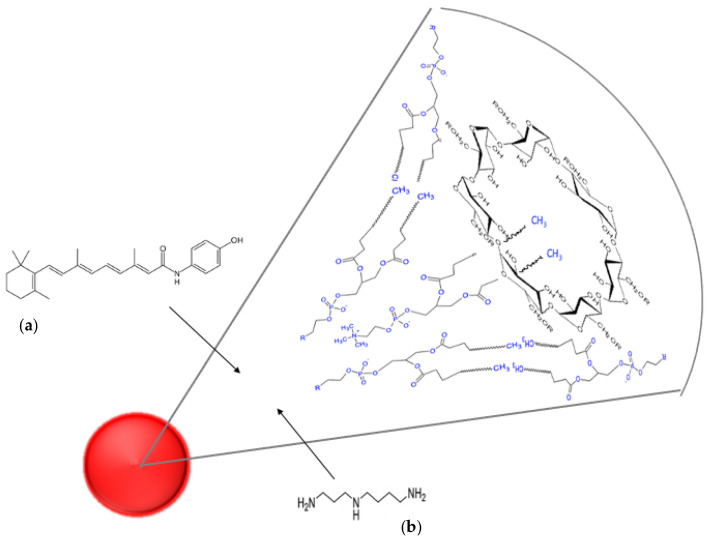
Schematic representation of the supramolecular organization of (**a**) Nanofenretinide and (**b**) Nanospermidine. Main constituents: fenretinide or spermidine, phospholipids and 2-hydroxypropyl beta cyclodextrin.

NS and NF were obtained by dispersion in PBS of the semisolid mixtures made of phospholipids, glyceryl tributyrate, 2-hydroxypropyl beta cyclodextrin, fenretinide, or spermidine. The amphiphilic character of the mixtures elicited supramolecular self-assembly in the aqueous environment, with the formation of nanomicelles and entrapment of fenretinide or spermidine in their inner matrix (Figure 2).

Drug loading, particle size, polydispersity, zeta potential, and stability to drug leakage were measured on NS and NF suspensions at 0.05 mg/mL in PBS containing 10% (*v*/*v*) HS to simulate the concentrations of the nanomicelles in the medium used for the cytotoxicity experiments. The results indicated that the nanomicelles were suitable for tumor accumulation by the Enhanced Permeability and Retention (EPR) effect [31,32,33,34,35] being their mean size under 300 nm and the polydispersity index lower than 0.2, which corresponds to a homogenous size distribution. The nanomicelles were characterized by a negative surface charge, as indicated by the zeta potential values (Table 1). 

NS had a lower zeta potential than NF in accordance with the alkaline character of the polyamine spermidine. Both NF and NS were characterized by stability to drug leakage, being the release lower than 20% of the drug loading after 24 h. This feature improves the nanoparticles’ ability to convey high drug amounts at the tumor site, avoiding uncontrolled drug release during their permanence in circulation. 

### 3.2. Effect of Nanofenretinide, Nanospermidine, and Naxitamab on Cell Viability

**Figure 3:**
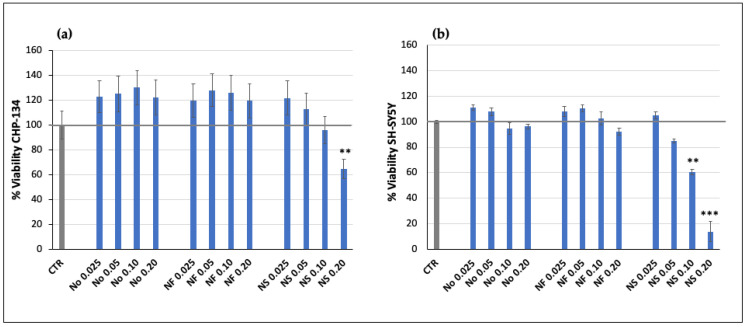
Relative viability of (**a**) CHP-134 and (**b**) SH-SY5Y cells treated with increasing concentrations of Nanofenretinide (NF) and Nanospermidine (NS) for 24 h in comparison with empty nanomicelles (No). Viability was evaluated by MTT assay and expressed as percentage versus control (100%) (mean ± SD, n = 6) (** *p* < 0.01, *** *p* < 0.001).

Treatment with NF at concentrations in the range 0.025–0.2 mg/mL corresponding to fenretinide concentrations 5–40 μM did not provide significant cytotoxicity in CHP-134 and SH-SY5Y (Figure 3). NS, in the same nanoparticle concentration range corresponding to 22–178 μM spermidine, decreased cell vitality in both cell lines. The effect was proportional to NS concentration and was higher in SH-SY5Y than CHP-134 (Figure 3). The empty nanomicelles, evaluated as a comparison, revealed no significant cytotoxic effects up to the higher concentration that was analyzed in both cell lines (Figure 3).

**Figure 4:**
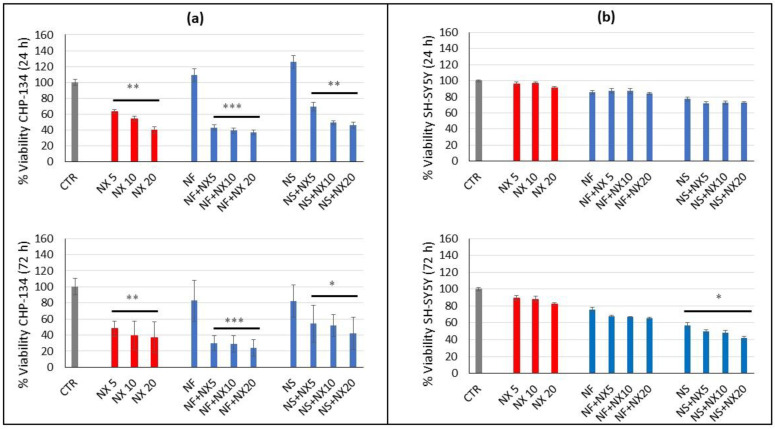
Relative viability of (**a**) CHP-134 and (**b**) SH-SY5Y cells treated with naxitamab at 5, 10, and 20 ug/mL and naxitamab in combination with 0.05 mg/mL Nanofenretinide (NF) or Nanospermidine (NS) for 24 h or 72 h. Viability was evaluated by MTT assay and expressed as percentage versus control (100%) (mean ± SD, n = 6) (* *p* < 0.05, ** *p* < 0.01, and *** *p* < 0.001).

Treatment with NX had a higher effect on CHP-134 than SH-SY5Y. Indeed in CHP-134 NX, the cell viability decreased to about 48%, 39%, and 37% after 72 h at 5, 10, and 20 μg/mL, respectively. In SH-SY5Y, the cell viability was about 89%, 88%, and 82% after 72 h treatment with NX at 5, 10, and 20 μg/mL, respectively (Figure 4). This was in accordance with the higher basal expression of GD2 in CHP-134 compared with SH-SY5Y cells, which are characterized by a very low basal GD2 expression. The effect of NX was also evaluated in combination with NF or NS at 0.05 mg/mL, since this nanomicelle concentration demonstrated minimal cytotoxicity at 24 h (Figure 3) in both cell lines and therefore, was suitable to evaluate the influence of NF and NS on NX activity. In addition, this nanomicelle concentration can be easily achieved in vivo as demonstrated in NB xenografts after a single NF administration [20]. The viability studies indicated that in CHP-134, NF plus NX decreased the cell viability more than the single components while NS plus NX did not appreciably influence the effect of NX on the cell viability (Figure 4).

**Figure 5:**
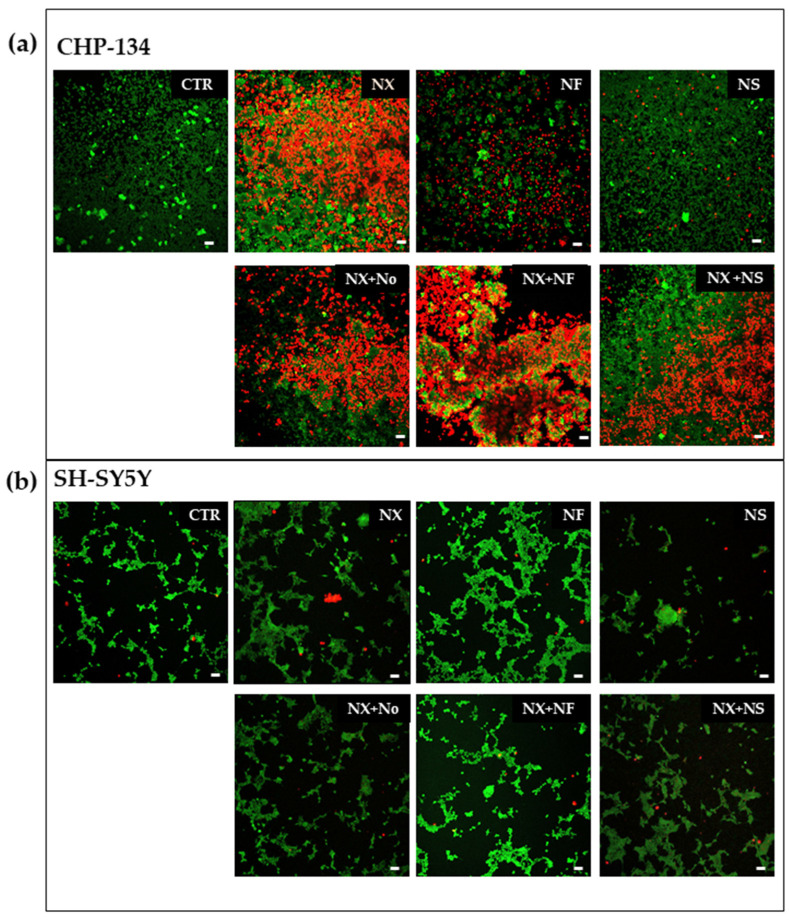
Fluorescence images of (**a**) CHP-134 cells and (**b**) SH-SY5Y cells stained with calcein and PI to show the live (green) and dead (red) cells after 72 h treatment with NX (20 μg/mL), NF, NS (0.05 mg/mL), NX in combination with NF, NX in combination with NS, and NX in combination with the empty nanomicelles (No). Photographs were taken at 10X magnification, bar = 10 μm.

These results are supported by the fluorescence microscopy images of CHP-134 showing an improvement of cell death by treatments with NF plus NX, but not with NS plus NX, with respect to the sole NX (Figure 5a). Moreover, treatments with empty nanomicelles plus NX did not show any difference with respect to the single NX treatment, thus indicating a lack of activity of the empty nanomicelles.

In SH-SY5Y cells, treatments with NF in combination with NX did not significantly decrease the cell viability with respect to the single components and treatment with NS in combination with NX provided minor effects (Figure 4).

The fluorescence microscopy images of SH-SY5Y did not show appreciable cell death by the treatments with NF plus NX, NS plus NX, or with the single components (Figure 5b). Taken together, these data suggest that a cytostatic effect is induced by NF and NS in SH-SY5Y without involvement of NX.

### 3.3. Quantitative Phase Imaging

**Figure 6:**
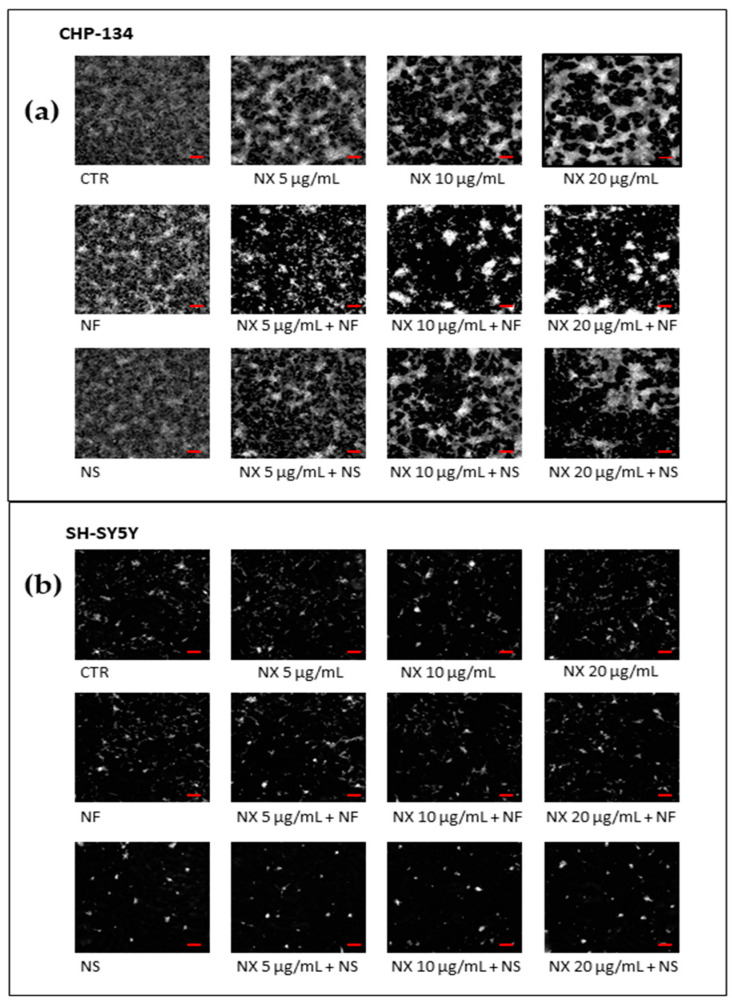
Representative images at 72 h of (**a**) CHP-134 and (**b**) SH-SY5Y cells treated with NX at 5, 10, and 20 μg/mL, or NF, NS (0.05 mg/mL), NX in combination with NF, and NX in combination with NS. Photographs were taken at 10X magnification, bar = 10 μm.

The images of the cells reported in Figure 6 were obtained after 72 h treatment with NF, NS, NX, NF plus NX, and NS plus NX. They confirmed the MTT results, indicating that the most evident cytotoxic effects were obtained with NF plus NX in CHP-134. The corresponding videos are reported as Supplementary Materials.

**Figure 7:**
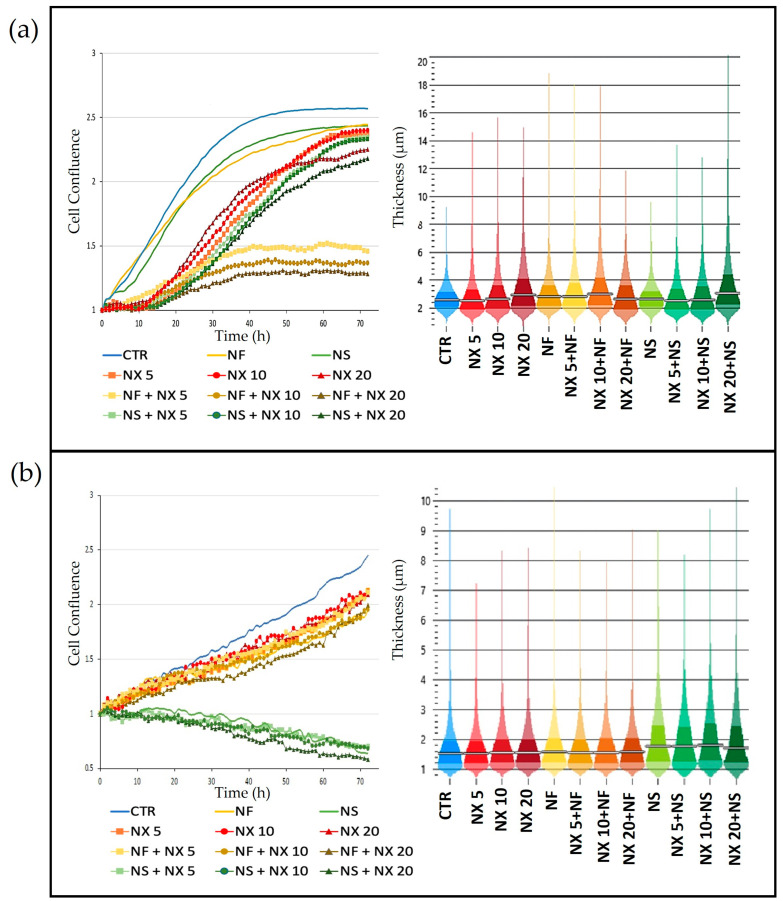
Cell confluence and thickness of (**a**) CHP-134 cells and (**b**) SH-SY5Y cells treated with NX at 5, 10, and 20 μg/mL, or NF, NS (0.05 mg/mL), NX in combination with NF, and NX in combination with NS.

Additionally, the trends of cell proliferation over time confirmed the MTT results. Indeed, a strong inhibition of cell confluence was observed in CHP-134 cells treated with NF plus NX. The inhibition was proportional to the NX concentration. Treatment with NS plus NX, on the contrary, did not significantly decrease cell confluence, with respect to single NX (Figure 7a). 

In SH-SY5Y, NF did not appreciably influence cell confluence with respect to NX. Treatment with NS and NS plus NX, on the contrary, strongly decreased the cell confluence over time, suggesting that the cells grew in aggregates as indicated by the increased cell thickness induced by treatment with NS and NS plus NX (Figure 7b).

**Figure 8:**
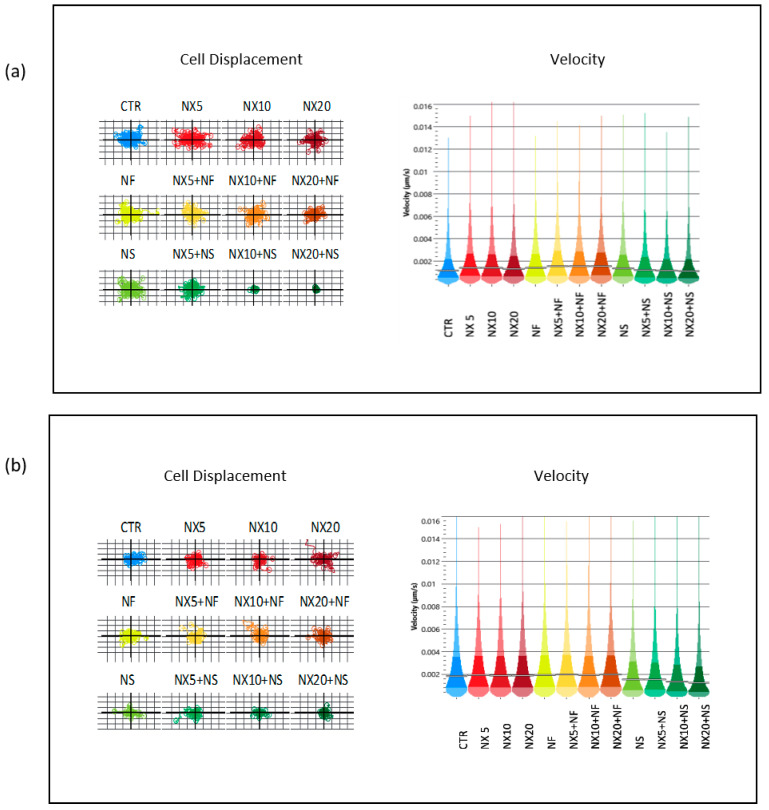
Cell displacement and instantaneous velocity of (**a**) CHP-134 cells and (**b**) SH-SY5Y cells treated with NX at 5, 10, and 20 μg/mL, or NF, NS (0.05 mg/mL), NX in combination with NF, and NX in combination with NS.

Cell motility is a very important feature in influencing the tumor outcome since it contributes to cancer invasion and metastasis [36,37,38]. Moreover, deregulation of this parameter is involved in several pathologies, such as autoimmune disorders, neurological diseases, and chronic inflammation [36]. In our study, taking advantage of the use of ptychography in a time lapse microscopy, we evaluated, by means of these non-invasive label-free imaging measurements, cell displacement and instantaneous velocity as measures of the cell ability to migrate from the original site after treatment. The results indicated that NS plus NX inhibited cell displacement and instantaneous velocity in both cell lines (Figure 8). The inhibition increased with increasing the antibody concentration.

### 3.4. GD2 Expression on Cell Surface

**Figure 9:**
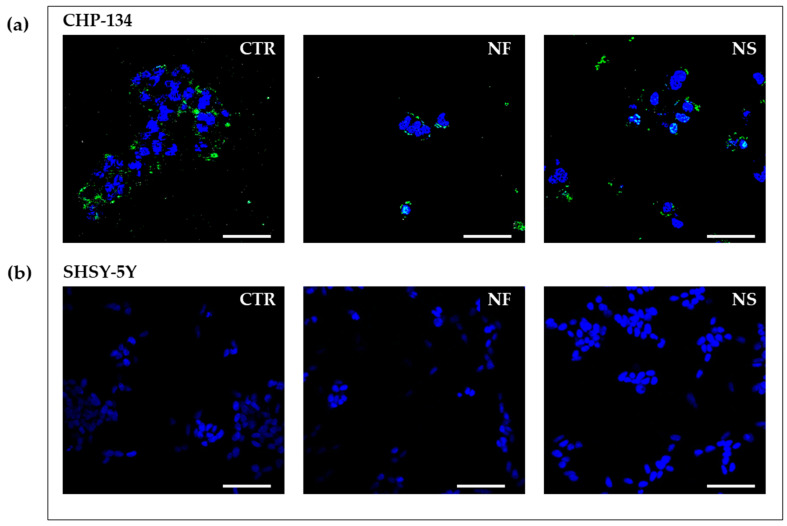
Confocal microscopy of (**a**) CHP-134 cells and (**b**) SH-SY5Y cells treated with NF or NS at 0.05 mg/mL for 24 h and subsequently exposed for 1 h to 20 μg/mL naxitamab-fluorescein conjugate (NX-F). Photographs were taken at 40X magnification, bar = 10 μm.

Cells treated with NS or NF for 24 h and subsequently added with NX-F were visualized by means of the confocal laser-scanning fluorescence microscopy. The images (Figure 9) showed fluorescence on the treated CHP-134 cells but also on the untreated controls, indicating that a high basal expression of GD2 is already present on these cells. The SH-SY5Y cells, on the contrary, did not show any detectable fluorescence after treatment. The lack of fluorescence on the controls indicates that these cells are characterized by very low expression of GD2.

**Figure 10:**
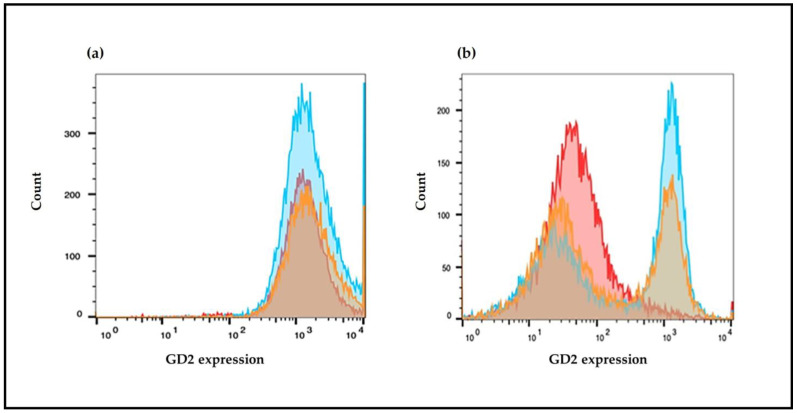
Flow cytometry analysis of GD2 expression in (**a**) CHP-134 cells and (**b**) SH-SY5Y cells treated with NF or NS (0.05 mg/mL) for 72 h and subsequently stained with NX-F (20 μg/mL). Fluorescence distribution of control cells stained with NX-F (red) and cells stained with NX-F after treatment with NF (blue) and NS (yellow), respectively.

Cells stained with NX-F were also analyzed by flow cytometry to quantitatively evaluate the expression of GD2. The results indicated that the CHP-134 cells were characterized by a high and uniform expression of GD2 on their surface (Figure 10a), while SH-SY5Y demonstrated a lower and heterogeneous basal expression of GD2 (Figure 10b). The treatment of CHP-134 with NF and NS induced about an 11–12% increase in fluorescence with respect to the control (Figure 10 and Table 2). Free fenretinide and free spermidine, on the contrary, did not increase fluorescence, but generated a negative trend (Table 2). These results indicated a favorable role of the nanomicelles in transporting the encapsulated drugs inside the cells with a consequent increase in intracellular drug concentrations at levels suitable to trigger specific responses. In SHSY-5Y cells, treatment with NF and NS increased fluorescence in a significant portion but not in all the cell population. The percentage of bright cells was around 40–50% and the fluorescence increase was approximately eight-fold when compared to the control. Treatments with free fenretinide and free spermidine provided similar results as the nanoencapsulated drugs.

## 4. Discussion

The antitumor activity of anti-GD2 antibodies is well-known and it relies on different mechanisms. The higher the GD2 expression, the higher the extent of the antibody linkage to the GD2 molecules and the intensity of the correlated response [1,2,3,4,5,6,7].

Many pre-clinical and clinical studies performed on neuroblastoma have largely demonstrated that the efficacy of anti-GD2 antibodies, such as naxitamab, mainly depends on the levels of GD2 expression on the tumor cell surface and the cell sensitivity to anti-GD2 mAbs effects. Therefore, new approaches to enhance the expression of GD2 on tumor cells or to increase the sensitivity of cells to the effects of anti-GD2 mAbs are actively sought to increase the efficacy of anti-GD2 mAbs treatments in anticancer therapy. 

In this work we have demonstrated that an increased GD2 expression can be obtained in NB cells by treatment with Nanofenretinide and Nanospermidine, which are two nanoformulations based on phospholipid nanomicelles containing fenretinide and spermidine, respectively [22].

In CHP-134 cells, characterized by high and homogeneous basal expression of GD2, Nanofenretinide and Nanospermidine induced a 10–12% increase in GD2 expression with respect to the control. On the contrary, treatment with free fenretinide and free spermidine at the same concentrations as Nanofenretinide and Nanospermidine generated a negative trend towards GD2 expression, indicating a favorable role of the nanomicelles in transporting the encapsulated drugs inside the cells, thus improving the intracellular concentrations of drugs at levels triggering specific responses. 

The increased expression of GD2 induced by Nanofenretinide and Nanospermidine was expected to provide an overall improved cytotoxicity by treatment with naxitamab in combination with the nanodrugs. However, the results indicated that only the combination of Nanofenretinide plus naxitamab provided an increased activity with respect to the single components, while Nanospermidine plus naxitamab did not provide an overall increased activity. 

This different behavior can be correlated to different mechanisms activated by Nanofenretinide and Nanospermidine inside the cells that can reinforce or weaken the cytotoxic effect triggered by the naxitamab linkage to GD2.

Nanofenretinide appeared to reinforce the activity of naxitamab. Indeed, Nanofenretinide plus naxitamab significantly decreased cell viability with respect to the single drugs. An explanation of the positive role of Nanofenretinide on naxitamab effect can be found in the inhibition of the PI3K/AKT/mTOR pathway that has been demonstrated for both fenretinide [39] and anti-GD2 mAbs [7] in NB cells. All the other mechanisms by which fenretinide has been proven to induce cell death in tumor cells may be supposed to promote cell sensitivity to naxitamab, rather than inducing cell death, when the drug is administered at sub-cytotoxic concentrations as in the present study.

Nanospermidine, on the contrary, did not significantly influence naxitamab cytotoxicity. Indeed, it did not provide a decrease in cell viability but slightly increased viability with respect to naxitamab administered as a single agent. However, a significant decrease in cell displacement and instantaneous velocity was observed with Nanospermidine plus naxitamab. The inhibition effect on cell motility increased with the increase in the naxitamab concentration. 

Nanospermidine had been previously demonstrated to induce cell death in NB cells by transporting supraphysiological concentrations of spermidine inside the cells with a consequent over-production of ROS [22]. The correlation between stress-activated pathways and cell sensitivity to anti-GD2 mAbs activity had been reported in the literature [23]. Anti-cancer drugs such as doxorubicin, topotecan, carboplatin, cisplatin, etoposide, and paclitaxel have been observed to increase anti-GD2 mAbs activity by activation of stress-correlated pathways such as JNK/SAPK or PERK that make the tumor cells more sensitive to the antibody effect [24,25]. Yet, the lack of the Nanospermidine effect on naxitamab cytotoxicity is probably related to the sub-cytotoxic concentrations of Nanospermidine used in the present study that did not provide a sufficient ROS increase to trigger the stress-activated pathways and promote cell sensitivity to naxitamab. However, the strong inhibition of cell displacement and instantaneous velocity suggests that the treatment with Nanospermidine plus naxitamab could inhibit the cell spread capacity.

The importance of motility inhibition of tumor cells is well-known. It represents an approach that can influence the outcome of treatments. Many therapeutic strategies are being evaluated to block tumor cell displacement with the aim to prevent cell spreading to sites that are distant from the primary tumors with the formation of metastases that are often un-accessible to drug distribution and represent the main cause of relapse.

As a comparison, we also evaluated the effect of Nanofenretinide and Nanospermidine on SH-SY5Y, a well-known cell line characterized by a low and heterogeneous basal expression of GD2. The heterogeneous GD2 expression is due to the presence of both undifferentiated and differentiated cell populations in SH-SY5Y, whose relative ratio can change upon treatment [40]. These characteristics make SH-SY5Y cells suitable to simulate the heterogeneous cell populations present in a tumor mass after chemotherapy.

Treatment with Nanofenretinide and Nanospermidine increased GD2 expression in a significant portion but not all of the SH-SY5Y cell populations. The percentage of cells with an increased GD2 expression was about 50% and the increase in GD2 expression was approximately eight-fold compared to the control. 

The combinations of Nanofenretinide and Nanospermidine with naxitamab provided cell viabilities similar to those obtained by single Nanofenretinide and Nanospermidine treatments, indicating that the concentrations of Nanofenretinide and Nanospermidine used in this study were not sufficient to produce a surface density of GD2 suitable to trigger an appreciable increase in naxitamab cytotoxicity. However, these results indicate the potential of Nanofenretinide and Nanospermidine to increase GD2 expression in refractory cells when used at higher concentrations or repeatedly. 

Moreover, this study demonstrates that the improvement of GD2 on NB cells does not always provide an increase in the cytotoxic effect of anti-GD2 mAbs, such as naxitamab, since the enhanced cytotoxicity, due to the GD2 increase, can be reinforced or weakened by the concomitant activity of co-administered drugs. However, the co-administered agents can as well provide other useful effects such as cell motility decrease.

Furthermore, the cytotoxic effect evaluated in vitro represents only a small part of the whole antitumor mechanism involving, in vivo, the activation of the immune cell-mediated responses such as ADCC and ADP. The in vitro studies can only provide indications on the antibody ability to induce cell cytotoxicity by a direct effect on the cells and by activation of CDC. Therefore, the increased naxitamab cytotoxicity that was induced, in vitro, by Nanofenretinide, can be expected to provide high antitumor effects in vivo due to the immune cells contribution. 

Even the minor effects provided by Nanospermidine that did not increase the cytotoxicity of naxitamab but decreased cell displacement can turn into a useful tool in vivo, where it can sum up to the effects of the immune cells.

Additionally, in NB cells that are expressing low basal GD2 levels, where Nanofenretinide and Nanospermidine induced GD2 improvement in a limited cell population, an increased antitumor effect can be expected in vivo, either by the presence of the immune cells or by repeated administrations of Nanofenretinide and Nanospermidine in combination with naxitamab, which will repeatedly target the sensitive population, thus reducing their burden.

## 5. Conclusions

Increased GD2 expression can be obtained in NB cells by treatment with Nanofenretinide and Nanospermidine. 

The improved GD2 expression can provide a decreased cell viability by co-treatment with Nanofenretinide plus naxitamab or a significant inhibition of cell motility by co-treatment with Nanospermidine plus naxitamab. 

Therefore, the use of naxitamab in combination with Nanofenretinide or Nanospermidine should be considered in NB therapy in order to improve the pharmacologic efficacy and clinical responses to anti-GD2 treatments. 

These results, obtained in vitro, are expected to further improve in in vivo settings, due to the contribution of the immune cell-mediated ADCC and ADP, representing key mechanisms in the overall antitumor activity of anti-GD2 mAbs as well as CAR-T and CAR-NK cells-based immunotherapies.

## Figures and Tables

**Table 1:** Physicochemical characteristics of Nanofenretinide (NF) and Nanospermidine (NS) in PBS at 0.05 mg/mL.

Nanomicelle Type	% Loading (*w*:*w*)	Mean Size (nm)	Polydispersity Index	Zeta Potential (mV)	% Drug Leakage (24 h)
NF	Fenretinide 8.03 ± 0.910	209.70 ± 2.41	0.18 ± 0.01	−25.4 6 ± 1.57	16.30 ± 2.79
NS	Spermidine 12.58 ± 1.95	254.63 ± 2.94	0.16 ± 0.02	−16.30 ± 0.41	13.60 ± 1.01

**Table 2:** NX-F staining of CHP-134 and SH-SY5Y cells to evaluate the fluorescence fold increase induced by treatment with NF or NS (0.05 mg/mL), free fenretinide (F), or free spermidine (S) at the same concentrations as contained in the nanomicelles (10 μM fenretinide, 44 μM spermidine). As a comparison, the cells were treated with empty nanomicelles (No).

	CHP-134		SH-SY5Y	
	% Bright Cells	Fluorescence Fold Increase with Respect to Control	% Bright Cells	Fluorescence Fold Increase with Respect to Control
F	100	−0.90	33.05	8.25
S	100	−0.88	52.65	8.08
NF	100	1.11	47.69	7.53
NS	100	1.16	39.53	7.70
No	100	−0.92	7.35	0.80

## Data Availability

The data presented in this study are available on request from the corresponding authors.

## Supplementary Materials

The following supporting information can be downloaded at: https://www.mdpi.com/article/10.3390/pharmaceutics15020648/s1, Figure S1: (a) Video from 0 to 72 h of CHP-134 cells treated with NX at 5, 10, 20 µg/mL, NF or NS (0.05 mg/mL), NX in combination with NF and NX in combination with NS; (b) Video from 0 to 72 h of SH-SY5Y cells treated with NX at 5, 10, 20 µg/mL, NF or NS (0.05 mg/mL), NX in combination with NF and NX in combination with NS.
