# Supplementary material for: Naxitamab Activity in Neuroblastoma Cells Is Enhanced by Nanofenretinide and Nanospermidine
Source: Pharmaceutics. 2023 Feb 15;15(2):648. doi: 10.3390/pharmaceutics15020648 (PMC9966923; doi:10.3390/pharmaceutics15020648)
Supplement: Supplementary file 1 [file pharmaceutics-15-00648-s001.zip › pharmaceutics-2193053-supplementary.pptx]

## Slide 1
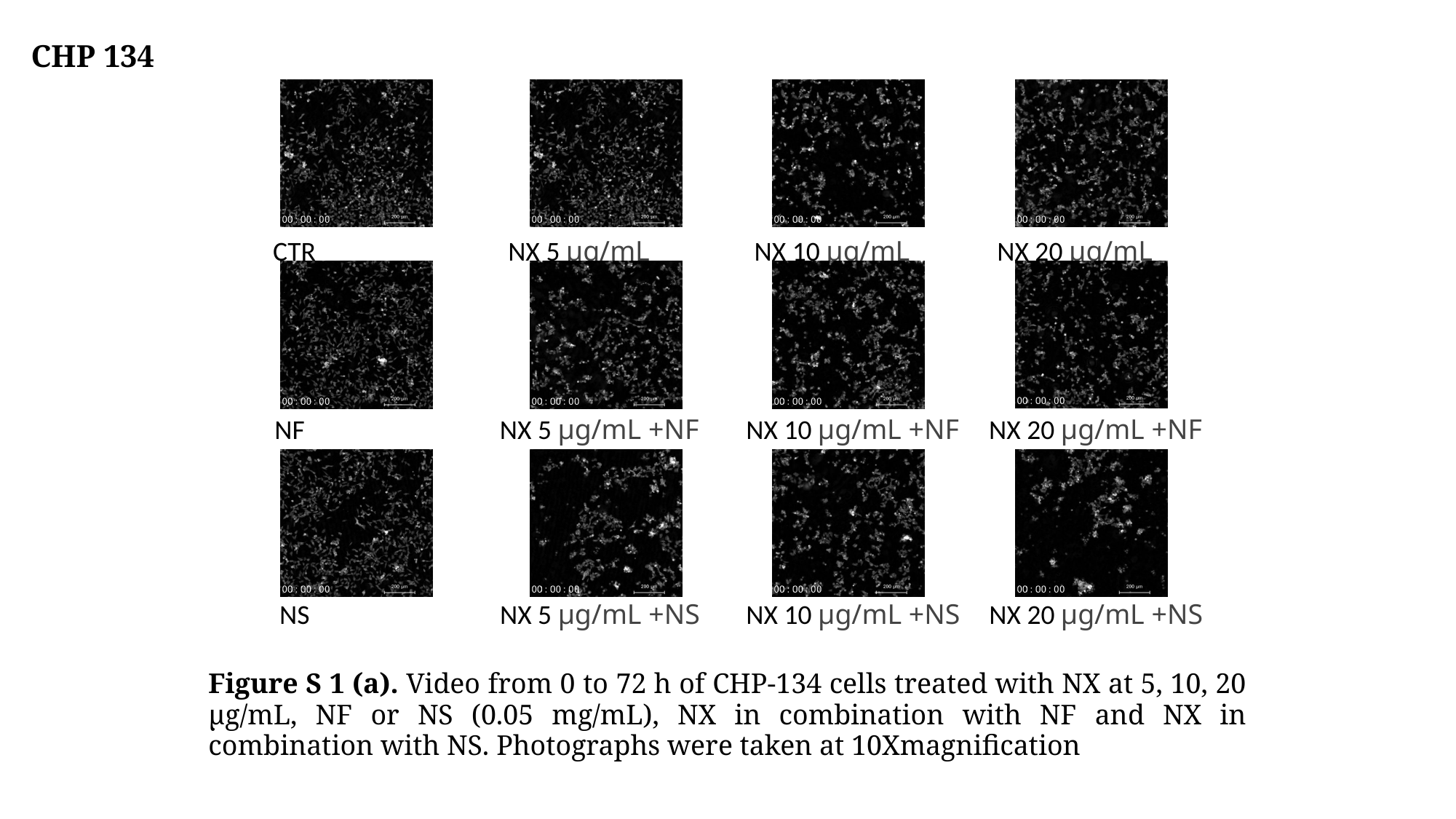

CHP 134
CTR
NX 5 μg/mL
NX 10 μg/mL
NX 20 μg/mL
NF
NX 5 μg/mL +NF
NX 10 μg/mL +NF
NX 20 μg/mL +NF
NS
NX 5 μg/mL +NS
NX 10 μg/mL +NS
NX 20 μg/mL +NS
Figure S 1 (a). Video from 0 to 72 h of CHP-134 cells treated with NX at 5, 10, 20 µg/mL, NF or NS (0.05 mg/mL), NX in combination with NF and NX in combination with NS. Photographs were taken at 10Xmagnification

## Slide 2
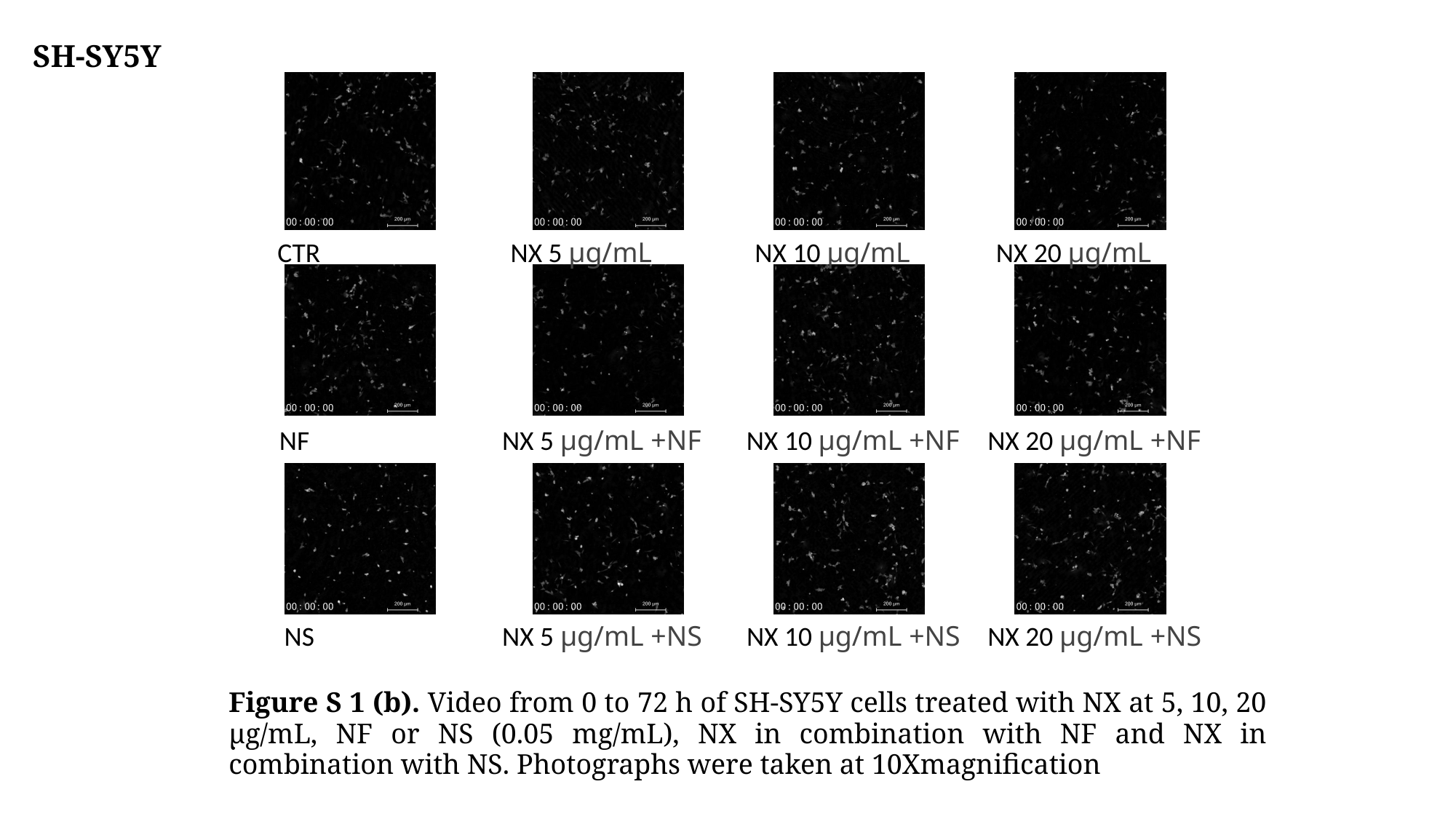

SH-SY5Y
CTR
NX 5 μg/mL
NX 10 μg/mL
NX 20 μg/mL
NF
NX 5 μg/mL +NF
NX 10 μg/mL +NF
NX 20 μg/mL +NF
NS
NX 5 μg/mL +NS
NX 10 μg/mL +NS
NX 20 μg/mL +NS
Figure S 1 (b). Video from 0 to 72 h of SH-SY5Y cells treated with NX at 5, 10, 20 µg/mL, NF or NS (0.05 mg/mL), NX in combination with NF and NX in combination with NS. Photographs were taken at 10Xmagnification
